# Supplementary material for: Defining and prioritizing modifiable risk factors towards the co-creation of a urinary incontinence self-management intervention for older men: A sequential multimethod study protocol
Source: PLoS One. 2024 Jul 25;19(7):e0305052. doi: 10.1371/journal.pone.0305052 (PMC11271917; doi:10.1371/journal.pone.0305052)
Supplement: S1 Appendix — (DOCX) [file pone.0305052.s001.docx]

**Appendix 1: Delphi survey**

**Demographic and practice characteristics**:

1. What is your specific occupation within the medical field? (select only one)

- Nurse practitioner
- Family Physician
- Geriatrician
- Urologist
- Other: ………………………………………

1. How many years have you been working in this area of expertise? ………………….
2. Your main affiliation is with what type of organization? (select only one)

- Higher education (e.g., college, university) or publicly funded research institution
- Government or arm’s length public administration agency (e.g., assessment, regulation, standards and norms, procurement, etc.)
- Healthcare facility (e.g., hospital, outpatient clinic, community health service, etc.)
- For-profit organization, professional consultant firm or privately funded research institution
- Charity, not-for-profit organization, non-governmental organization (NGO) or multilateral body (e.g., WHO, United Nations)
- Other: ………………………………………………………………….

1. In which country are you primarily located?.............................................
2. To which gender identity do you self-identify? (select only one)

- Female gender
- Male gender
- Non-binary
- Prefer not to respond
- Other: ……………………………………

1. What is your age (years)?

- Less than or equal to 30
- 31-40
- 41-50
- 51-60
- More than 60
- Prefer not to respond

**Item list for round 1 of the Delphi survey (Risk factors identified through a scoping review** [33]**)**

Instruction: Below is a list of evidence-synthesized risk factors for UI in older men. Please answer Yes or No to the stem questions regarding each factor and justify your choice, if applicable.

| **Factors** | **Is this risk factor in the development of UI in older men important**? | | **In your opinion, is it amenable to self-management?** | | Comments/justifications for your choice |
| --- | --- | --- | --- | --- | --- |
|  | Yes | No | Yes | No |  |
| **Demographic risk factors** |  |  |  |  |  |
| Age/Increasing age/Advanced age, Occupation, Race/ethnicity, Residential area (urban dwelling) |  |  |  |  |  |
| **Environmental risk factors** |  |  |  |  |  |
| Poor lighting, Cold, Nursing home/ Institutionalization, Early bedtime, Physical restraints, Use of cot-sides/bedrails, Lack of commode, Reliance on draw sheets and pads, Call bells forgotten, Sub-optimal nursing, Commode at improper height, Bathroom distance, Other environmental barriers |  |  |  |  |  |
| **Behavioral risk factors** |  |  |  |  |  |
| Tobacco smoking |  |  |  |  |  |
| Alcohol use |  |  |  |  |  |
| Caffeine intake |  |  |  |  |  |
| Intake of bladder irritants (carbonated beverages, citrus, artificial sweeteners) |  |  |  |  |  |
| **Physiological risk factors and age-related physiological changes** |  |  |  |  |  |
| Decreased maximum grip strength |  |  |  |  |  |
| *Age-related urinary tract functional decline*  Decline in renal function, Decreased functional bladder capacity/Increased post void residual volume, Decreased awareness of bladder filling, Decreased efficiency of bladder emptying, Age-related declined urethral function, Increase in nocturnal sodium and fluid excretion/Increased nighttime urine production |  |  |  |  |  |
| **Medical factors/diseases** |  |  |  |  |  |
| Increased BMI/overweight/ obesity, Greater waist circumference, Increased fat mass |  |  |  |  |  |
| Heart disease |  |  |  |  |  |
| BPH/Prostate problems |  |  |  |  |  |
| Diabetes mellitus |  |  |  |  |  |
| Depression/ Depressive mood/ symptoms |  |  |  |  |  |
| Constipation/fecal impaction |  |  |  |  |  |
| Brain injury |  |  |  |  |  |
| Anxiety |  |  |  |  |  |
| Hypertension |  |  |  |  |  |
| Voiding symptoms |  |  |  |  |  |
| UTI/Chronic UTI |  |  |  |  |  |
| Cognitive impairment, Dementia |  |  |  |  |  |
| Chronic diarrhea |  |  |  |  |  |
| Comorbid conditions/increased comorbidity |  |  |  |  |  |
| Prostate cancer, Bladder cancer |  |  |  |  |  |
| Insomnia/sleep disorder |  |  |  |  |  |
| Heart Failure |  |  |  |  |  |
| Urethral stricture |  |  |  |  |  |
| *Nervous system disorders*  Stroke/Prior Stroke, Parkinson's disease, Spinal cord compression/injury/ lesion, Multiple sclerosis, Normal pressure hydrocephalus, Cervical spondylotic myelopathy, Other chronic neurological diseases |  |  |  |  |  |
| Feacal incontinence |  |  |  |  |  |
| Chronic cough |  |  |  |  |  |
| Poor vision |  |  |  |  |  |
| Delirium |  |  |  |  |  |
| Hip fracture, Foot and ankle problems |  |  |  |  |  |
| Kidney disease |  |  |  |  |  |
| **Other factors** | | | |  |  |
| *Lower urinary tract-related factors*: Detrusor overactivity, DHIC (Detrusor Hyperactivity with Impaired Contractile Function), Detrusor underactivity/Underactive bladder, Detrusor sphincter dyssynergia (DSD), Atonic bladder |  |  |  |  |  |
| *Physical functioning-related factors* Limitation in physical function/ADL disability/low composite physical performance score |  |  |  |  |  |
| *Physical functioning-related factors* Immobility/Impaired mobility/unable to stand/ Reduced frequency and ease of getting out of the house |  |  |  |  |  |
| Polyuria/Nocturnal polyuria |  |  |  |  |  |
| Bladder outlet obstruction |  |  |  |  |  |
| *Medications* Medications/Polypharmacy, Diuretics, CNS depressants, Alpha-antagonists and agonists, Narcotic analgesics, Anticholinergics, Hypnotics/sedatives |  |  |  |  |  |
| Low serum testosterone |  |  |  |  |  |
| *Structural factors*  Myosteatosis (Low average total psoas density), Low obturator internus muscle thickness, Short membranous urethral length |  |  |  |  |  |
| Poor sleep quality/ sleep disturbance |  |  |  |  |  |
| Self-perception of health |  |  |  |  |  |
| Frailty |  |  |  |  |  |
| Incontinence less frequent than monthly |  |  |  |  |  |
| *Factors related to prostate cancer therapy*  Prostatectomy, Post-radiation therapy/Preoperative radiotherapy, Androgen deprivation therapy for Prostate Ca (PCa), Observation/watchful waiting in PCa, Prostatectomy-related neural injury/ Pudendal nerve injury, Ischemia during surgery, Surgical technique, Sphincter injury, Scar tissue immobilizing the sphincter |  |  |  |  |  |
