# Supplementary material for: Defining and prioritizing modifiable risk factors towards the co-creation of a urinary incontinence self-management intervention for older men: A sequential multimethod study protocol
Source: PLoS One. 2024 Jul 25;19(7):e0305052. doi: 10.1371/journal.pone.0305052 (PMC11271917; doi:10.1371/journal.pone.0305052)
Supplement: S2 Appendix — (DOCX) [file pone.0305052.s002.docx]

**Appendix 2: Older men’s survey questionnaire**

**Eligibility/screening questions**

| Age 65 or older  Yes  No |
| --- |
| How often do you experience urinary leakage?  1. Less than once a month  2. A few times a month  3. A few times a week  4. Every day and/or night  5. Never |
| How much urine do you lose each time?  1. No urine leakage  2. Drops  3. Small splashes  4. More |

**Survey questionnaire**

| **A: Sociodemographic and clinical characteristics** |
| --- |
| **Date of birth: Age**: |
| **Educational attainment** |
| <High school |
| High school |
| Postsecondary education |
| **Employment status** |
| Employed |
| Unemployed |
| **Socio-economic status:** How would you rate your family’s socioeconomic status/how would you describe your family’s financial situation? |
| Low |
| Middle-low |
| Middle |
| Middle-high |
| High |
| **Racial/Ethnic category of participants** |
| White |
| Black or African American |
| Asian |
| Hispanic or Latino |
| Native Hawaiian or Other Pacific Islander |
| **General health status** |
| Excellent |
| Very good |
| Good |
| Fair |
| Poor |
| **Duration of urinary incontinence symptoms, years** |
| <1 |
| 1-5 |
| >5 |
| **Frequency of UI episodes** |
| ≤1 time/week |
| 2–3 times/week |
| 1 time/day |
| Several times a day |
| All the time |
| **Amount of urine loss per episode** |
| A small amount |
| A moderate amount |
| A large amount |
| **Number of pads used per day** |
| None |
| 1 |
| 2 |
| 3 |
| ≥4 |
| **Average number of UI episodes per day recorded on the voiding diary**: |
| **Previous consultation for UI (Yes/No)** |
| **UI specialist diagnosis** |
| Stress UI |
| Urgency UI |
| Mixed UI |
| Overflow UI |
| Functional UI |
| None |
| **UI treatment** |
| Behavioral |
| Pharmacologic |
| Surgical |
| None |

**Incontinence Quality of Life (I-QOL) Instrument**

|  | | Extremely | Quite a bit | Moderately | A little | Not at all |
| --- | --- | --- | --- | --- | --- | --- |
| 1. | I worry about not being able to get to the toilet on time. |  |  |  |  |  |
| 2. | I worry about coughing and sneezing |  |  |  |  |  |
| 3. | I have to be careful about standing up after sitting down |  |  |  |  |  |
| 4. | I worry where the toilets are in new places |  |  |  |  |  |
| 5. | I feel depressed |  |  |  |  |  |
| 6. | I don’t feel free to leave my home for long periods of time |  |  |  |  |  |
| 7. | I feel frustrated because my UI prevents me from doing what I want |  |  |  |  |  |
| 8. | I worry about others smelling urine on me |  |  |  |  |  |
| 9. | Incontinence is always on my mind |  |  |  |  |  |
| 10. | It’s important for me to make frequent trips to the toilet |  |  |  |  |  |
| 11. | Because of my incontinence, it is important to plan every detail in advance |  |  |  |  |  |
| 12. | I worry about my incontinence getting worse as I grow older |  |  |  |  |  |
| 13. | I have a hard time getting a good night’s sleep |  |  |  |  |  |
| 14. | I worry about being embarrassed or humiliated because of my incontinence |  |  |  |  |  |
| 15. | My incontinence makes me feel like I’m not a healthy person |  |  |  |  |  |
| 16. | My UI makes me feel helpless |  |  |  |  |  |
| 17. | I get less enjoyment out of life because of my UI |  |  |  |  |  |
| 18. | I worry about wetting myself |  |  |  |  |  |
| 19. | I feel like I have no control over my bladder |  |  |  |  |  |
| 20. | I have to watch what I drink |  |  |  |  |  |
| 21. | My UI limits my choice of clothing |  |  |  |  |  |
| 22. | I worry about having sex |  |  |  |  |  |

**Subscale structure**:

Avoidance and limiting behavior: items 1, 2, 3, 4, 10, 11, 13, and 20

Psychosocial impacts: items 5, 6, 7, 9, 15, 16, 17, 21, and 22

Social embarrassment: items 8, 12, 14, 18 and 19

**Section B: Geriatric Self-Efficacy for UI Index (GSE-UI)**

0 1 2 3 4 5 6 7 8 9 10

Not confident Moderately confident extremely confident

| **Question** | | **Score out of 10** |
| --- | --- | --- |
| How confident are you that you can hold in your urine……….. | | |
| 1. | when you are at home and have to go to the bathroom? |  |
| 2. | when you are away from home? |  |
| 3. | long enough to get to the bathroom in time during the night? |  |
| 4. | for at least 20 minutes when you feel the urge? |  |
| 5. | when coughing? |  |
| 6. | when sneezing? |  |
| 7. | when laughing? |  |
| 8. | when you are nervous? |  |
| 9. | visit places where you may have difficulty locating the  bathroom? |  |
| 10. | go out on social outings without worrying about urine loss? |  |
| 11. | prevent urine loss without relying on pads or protection when  you are at home? |  |
| 12. | prevent urine loss without relying on pads or protection when you are out? |  |
| **Total Score** | |  |

**Section C: Expert-identified modifiable risk factors for UI in older men (subject to findings from the Delphi study)**

| Here is a list of things that make it more likely that a person will have symptoms of urinary incontinence (accidentally leaking pee/urine). If making changes to these things in your life may help to keep your symptoms from getting worse, please tell us how you feel about your willingness and ability to make a change. | | | | | |
| --- | --- | --- | --- | --- | --- |
| 1. **Willingness: changes you do not or do want to make**  For the things in the list below, please choose the answer that is closest to how you feel about your willingness to make a change. | | | | | |
| List | **Response** | | | | |
|  | I DO NOT want to make a change | I might be willing to make a change | I am pretty sure that I want to make a change | I definitely want to make a change | Does not apply to me |
| x. …………………… |  |  |  |  |  |
| y. …………………… |  |  |  |  |  |
| z…………………….. |  |  |  |  |  |
| 2. **Capability: changes you know you cannot or can make**  For the things in the list below, please choose the answer that is closest to how you feel about your ability to make a change. | | | | | |
| List | **Response** | | | | |
|  | I am NOT able to make a change | I might be able to make a change | I am pretty sure that I am able to make a change | I am definitely able to make a change | Does not apply to me |
| x. …………………… |  |  |  |  |  |
| y. …………………… |  |  |  |  |  |
| z. …………………… |  |  |  |  |  |

| One of the goals of our study is to develop some resources to help men like you to manage their condition. What kind of resource package would you be most likely to use?  1. A face-to-face educational workshop  2. An app on your phone or tablet for private use  3. A combination of face-to-face workshop and an app  4. Other (please specify) …………………………………………………………  . |
| --- |
